# Towards Resilient Healthcare Systems: A Framework for Crisis Management

**DOI:** 10.3390/ijerph21030286

**Published:** 2024-02-29

**Authors:** Seyedeh Gelareh Emami, Valentina Lorenzoni, Giuseppe Turchetti

**Affiliations:** Institute of Management, Scuola Superiore Sant’Anna, 56127 Pisa, Italy; valentina.lorenzoni@santannapisa.it (V.L.); giuseppe.turchetti@santannapisa.it (G.T.)

**Keywords:** resilience, crisis management, healthcare system

## Abstract

This study addresses the crucial need for resilient healthcare systems, highlighted by recent global health emergencies such as the Ebola and COVID-19 crises. It identifies a significant gap in the current literature: a lack of practical, actionable frameworks for healthcare resilience. To bridge this gap, the research introduces an innovative framework that blends theoretical resilience concepts with heuristic approaches. This framework, rooted in the principles of monitoring, anticipation, recognition, and learning, is designed to enhance the crisis management capabilities of healthcare systems. The methodology involves a comprehensive literature review, combined with heuristic methods, culminating in a framework that is both academically sound and practically applicable. This framework guides healthcare systems through various stages of crisis management, including data collection, situation analysis, risk anticipation, and response evaluation. It provides a holistic approach to enhancing resilience in healthcare settings. Overall, this paper makes a significant contribution to the field of healthcare system resilience, offering a strategic blueprint for improved crisis response and recovery. It marks an important advancement in aligning theoretical resilience concepts with practical implementation strategies, essential for tackling current and future healthcare challenges.

## 1. Introduction

In modern society, healthcare systems act as pivotal pillars of community well-being and social infrastructure. They serve crucial roles not only in diagnosing and treating illness but also in preventive care, public health initiatives, and even economic stabilization. The significance of a well-functioning healthcare system becomes significantly pronounced in times of crisis, ranging from outbreaks of infectious diseases to natural disasters and human-induced emergencies. During these crises, a healthcare system is evaluated on two primary fronts: its immediate response capabilities, which include prompt medical intervention and resource allocation, and its resilience in the aftermath of the crisis, characterized by its ability to adapt, recover, and improve.

The necessity for resilient healthcare systems has been brought into sharp focus by recent global health emergencies. Events like the 2014 Ebola outbreak in West Africa and the ongoing COVID-19 pandemic that started in 2019 have not only stretched healthcare infrastructures to their limits but have also highlighted gaps and shortcomings that can have ripple effects far beyond the walls of hospitals and clinics. These gaps affect public health outcomes and can even influence economic stability at both the community and national levels. In light of these challenges, there is a compelling, urgent need to prioritize and bolster healthcare systems’ resilience, paving the way for a more robust, adaptive, and effective crisis management infrastructure.

A considerable amount of scholarly research has been dedicated to understanding and defining resilience in healthcare settings. These studies aim to identify the main characteristics of a resilient healthcare system, offering both empirical evidence and theoretical frameworks to explain how resilience operates and how it can be strengthened. The discourse surrounding healthcare resilience addresses the multi-dimensional challenges of implementing changes in various healthcare contexts, from rural clinics to sprawling urban hospital networks.

However, despite the extensive scholarly contributions, the current literature still exhibits several significant gaps. Most notably, there is a pronounced disconnect between theoretical rigor and practical applicability. Many existing frameworks for healthcare resilience offer nuanced insights but fall short of providing actionable guidelines that can be broadly implemented across diverse healthcare systems. This shortfall leads to an essential question that remains largely unexplored: “How can we effectively translate the theoretical understanding of healthcare resilience into a pragmatic, operational framework that is adaptable to various healthcare settings?”

This study employs crisis management plans to integrate a conceptual and heuristic framework to address this question. The principal objective of this research is to develop a robust framework that integrates theoretical knowledge with practical actionability. The framework aims to guide healthcare systems in effective crisis management and enhance their resilience in the face of future challenges by offering an adaptable approach for diverse healthcare environments with various features and needs.

By interrogating this pivotal gap, the investigation seeks to reconcile theoretical constructs with applied methodologies, thereby facilitating avenues for subsequent scholarly discourse and pragmatic interventions that are essential for fortifying healthcare systems.

## 2. Literature Review

### 2.1. Crisis Management and Resilience

The initial stage of scholarly investigation into resilience within healthcare systems necessitates a nuanced exploration of foundational elements. This involves a rigorous examination of the constructs of ‘crisis’ and ‘shock,’ which frequently serve as catalysts that challenge the resilience of healthcare systems. Not only do these constructs symbolize disruptive events, but they also function as benchmarks for assessing the robustness and adaptability of healthcare infrastructures. A ‘crisis’ is defined as a period of heightened difficulty, danger, or uncertainty, often precipitated by unforeseen events that disrupt the standard operational frameworks within healthcare settings. ‘Shocks,’ on the other hand, are delineated as either acute or chronic based on their temporal characteristics. The preponderance of academic literature on healthcare system resilience has predominantly focused on acute shocks, which are typically characterized by their sudden onset and shorter impact duration. Chronic shocks are those that persist over an extended timeframe, thereby posing distinct challenges to healthcare resilience [1]. Beyond the confines of healthcare, the discipline of crisis management has been a subject of scholarly investigation for numerous years and has found applicability across a broad spectrum of fields. Its principles and methodologies have been adapted and integrated into various scientific domains, underscoring its universal relevance and utility. In the context of healthcare systems, the adoption of crisis management frameworks thus represents an evolutionary step in fortifying resilience, benefiting from a wealth of interdisciplinary knowledge and best practices. A crisis management system is a structured approach involving policies, procedures, and actions designed to identify, assess, and manage risks and crises across various settings. It encompasses planning, communication, response coordination, and evaluation mechanisms to minimize impacts and enable an efficient recovery from disruptive events [2]. Given the pervasive utility of crisis management systems across various domains, their role assumes pronounced importance in the context of healthcare systems, which constitute critical infrastructures. Accordingly, substantial scholarly attention has been devoted to incorporating crisis management paradigms within healthcare settings in extant academic discourse [3].

The concept of resilience serves as a seminal augmentation to conventional crisis management paradigms, transitioning them from solely reactive modalities to more encompassing, anticipatory frameworks. By imbuing an adaptive, forward-looking dimension into the procedural fabric of crisis management, resilience transcends the immediate ambit of reactive measures and short-term recovery. It accentuates the system’s proactive faculties to anticipate, adapt, and recuperate from adversarial events, thus broadening the epistemological scope of crisis management to envelop considerations of long-term sustainability and systemic robustness. This confluence of immediate responsiveness and enduring resilience has garnered increasing recognition in scholastic discourse, thereby affirming its indelible significance as an integral component of a multi-faceted and efficacious approach to crisis management [4].

### 2.2. Healthcare System Resilience

The notion of resilience in healthcare systems, although extant for an extended period, witnessed an unprecedented surge in academic and practical relevance following the Ebola outbreak in West Africa. This catastrophic event served as a catalyst, compelling the global health governance structures to reframe resilience as an exigent, specialized paradigm indispensable for the fortification of health systems against an array of shocks, both acute and chronic [5,6]. Notwithstanding its ascendancy in scholarly and practical domains, the term ‘resilience’ has been characterized by a degree of terminological ambiguity, owing to the absence of a singular, universally accepted definition within the extant academic literature. The establishment of a coherent definitional framework is, therefore, not merely an academic exercise but a prerequisite for the development of a unified conceptual schema that can underpin resilience-oriented strategies and interventions.

As documented in peer-reviewed publications, the lexicon pertinent to the conceptual realm of resilience thinking within the domain of healthcare research has been taxonomically stratified into four cardinal categories: entities, intrinsic qualities, operative actions, and spheres of concern.

Predominantly within the existing body of scholarly work, resilience is variously conceptualized as either a characteristic [7], an ability [8], or a capacity [9,10,11]. These conceptions are applied across a heterogeneous array of entities, including but not limited to communities [12], individual [13] subjects, organizations [14,15], demographic cohorts [15,16], and infrastructure systems [13], each exhibiting varying degrees of resilience in the face of crisis events. Furthermore, in the definition of resilience in this context, it is mentioned that the main actions to achieve the same function [5,6,17] or control [10,14] over a shock are adaption [6,10], absorption [10], preparation [1,14,18], anticipation [7,19], transformation [16], response [17,20], and recovery [17].

The World Health Organization delineates system-level resilience as “the capacity of the health system to absorb, adapt, and change from the shock to maintain the system’s function” [21]. This definitional construct constitutes the foundational bedrock of our analytical framework. Within the purview of this investigation, healthcare resilience is conceptualized as a modality that facilitates crisis management by augmenting the system’s capacities to withstand shocks, all the while preserving its functional integrity.

### 2.3. Frameworks of Resilience in the Healthcare System

In contemporary academic discourse, a multitude of frameworks has been advanced to elucidate the nature of resilience in the healthcare sector. These scholarly contributions seek not only to provide a comprehensive understanding of the underlying mechanisms that bolster resilience but also to propose interventions and strategies that can further enhance the robustness and adaptability of healthcare systems in the face of adversity. A central theme consistently evident across these scholarly articles is the multi-dimensional nature of resilience; however, notable divergences arise in their specific focal points, methodological choices, and interpretative approaches.

For instance, some articles focused on healthcare governance [22], wherein systems thinking and complexity theories are synergistically integrated to develop frameworks tailored to bolster the resilience of entire healthcare infrastructures. Such frameworks predominantly dig into policy dynamics and the ramifications of governance architectures, underscoring the necessity of a macro-level perspective.

Diverging in its approach, Zhong et al. [23] shifted the lens to the organizational dimension, specifically targeting individual hospital settings. The proposed framework within this article utilizes benchmarks such as robustness, redundancy, and rapidity to assess the resilience of hospital operations amidst acute crises. While this scope might appear more circumscribed relative to previous ones, it provides an intricate view invaluable in the context of emergency response scenarios.

Some other scholars have navigated the realm of clinical perspectives, centering on patient-oriented frameworks. However, distinctions in their methodological approaches are evident. Agostini et al. offered a framework grounded in clinical outcomes and patient satisfaction indices [24]. In contrast, Förster et al. [25] accentuated the pivotal role of clinical leadership, effective communication, and the innovative integration of technology in bolstering resilience. Specifically, they advocated for a framework that synergistically melds telemedicine and electronic health records to amplify clinical resilience.

Drawing parallels with the thematic intricacies observed in previous studies, some other articles have delved into the realm of organizational psychology, explicitly exploring the psychological dimensions of healthcare system resilience. Even though these articles have mainly underscored the pivotal role of emotional intelligence and workplace culture in fortifying resilience, nuances in their emphasis are discernible [26,27]. Morse et al. prioritized the facets of interpersonal relationships and self-awareness [26], whereas Haraldseid-Driftland et al. critically engaged with organizational values and ethical considerations [27].

Complementing the specialized lenses of earlier studies, other studies have emerged at the nexus of diverse focal areas. Anderson et al. introduced a hybrid framework, interweaving system-level considerations with individual behaviors and organizational culture nuances [28]. By aiming for an encompassing perspective, it assimilates elements from multifarious frameworks found in the academic discourse.

Despite the wealth of insights these articles offer into healthcare resilience from varying perspectives—ranging from organizational psychology to system-level considerations—the literature appears to have notable gaps. Most of these frameworks adopt a predominantly theoretical standpoint. However, there is a conspicuous absence in the literature of a framework that is not only comprehensive and integrative but also future-oriented. There is a palpable need for research that holistically integrates the various dimensions into a comprehensive framework, ensuring both its present relevance and adaptability to future healthcare challenges.

In this study, we are motivated by two overarching aims. Firstly, by incorporating crisis management strategies, we seek to develop and implement a procedural framework tailored to the healthcare system. This framework is anticipated to streamline the deployment of reactive solutions, fostering an environment conducive to swift short-term recovery. Concurrently, our second aim is oriented towards the future: emphasizing the enhancement of the healthcare system’s proactive capabilities. By doing so, we aspire to cultivate resilience in the face of both imminent challenges and prospective shifts. Ultimately, our goal is to propose a comprehensive solution, one characterized by adaptability, ensuring it aligns seamlessly with the unique intricacies of each healthcare system.

## 3. Methodology

In our endeavor to create an optimal resilience framework for healthcare systems, we recognized the imperative need to amalgamate profound insights from the existing literature with the practical solutions offered by heuristics. These heuristics, characterized as algorithmic strategies or ‘rules of thumb’, simplify complex decision-making processes—a quality especially relevant in the dynamic healthcare environment. This strategic synthesis bridges in-depth scholarly knowledge with real-world applicability, aiming to offer a comprehensive yet actionable approach to resilience enhancement in healthcare settings.

Heuristic frameworks have been demonstrated to offer significant practical value, as evidenced by distinct research findings, in the realm of healthcare. For instance, a study highlights the utilization of heuristics in clinical settings, where healthcare professionals employ mental simulations and heuristics to effectively manage complex decision-making processes. This approach significantly reduces the cognitive load, allowing for more efficient and focused decision-making. It exemplifies how in scenarios of high uncertainty and complexity, clinicians can streamline their thought processes to prioritize the most critical information, leading to improved outcomes [29].

Another piece of research emphasizes the advantages of using simplified decision-making heuristics over traditional, information-intensive analysis methods in medical diagnoses. By focusing on a few key predictors, these heuristics enable both physicians and patients to make better-informed decisions, often with greater speed and less resource expenditure. This method proves particularly effective in cases where too much information can lead to analysis paralysis or when the rapidity of decision-making is paramount. The findings suggest that in certain healthcare contexts, less can indeed be more, offering a counterintuitive yet highly effective approach to decision-making [30].

The process of constructing conceptual frameworks involves a meticulous examination of peer-reviewed articles, facilitating the distillation of common themes, patterns, and recommendations. For this section, we embarked on a comprehensive scoping literature review focusing on the literature pertinent to resilience, through which we examined 259 articles [31]. Our objective was to identify parameters that would facilitate decision-makers in formulating decisions that contribute to augmenting resilience in times of shocks. Notably, these frameworks are known for their comprehensive theoretical insights. However, they may not inherently provide practical implementation steps, often residing predominantly within the theoretical realm.

In contrast, the heuristic framework was deliberately crafted to provide straightforward, actionable guidelines. This design ensures that healthcare professionals can enhance resilience without becoming entangled in dense theoretical nuances. By thoughtfully integrating heuristic principles with the broader themes and insights drawn from the literature, our integrated framework aspires to possess both academic rigor and practical utility. The research process concluded with feedback sessions from key stakeholders within healthcare systems and pilot applications of the framework, refining our model to ensure its adaptability and relevance in enhancing healthcare resilience.

In our methodology, we emphasized engaging with diverse healthcare stakeholders to ensure our framework’s relevance and applicability. Selection was based on their expertise and role, including frontline workers, administrators, policymakers, and patient advocates, to capture a wide-ranging perspective on healthcare resilience needs.

Initially, we conducted structured feedback sessions, presenting stakeholders with the preliminary framework. These sessions aimed to elicit in-depth critiques, suggestions, and potential enhancements, encouraging stakeholders to assess the framework’s comprehensiveness, real-world alignment, and integration ease into current healthcare processes. Following feedback, we piloted the framework in select healthcare settings, particularly within the European Reference Network (ERN) for rare diseases, leveraging its collaborative network for knowledge sharing and treatment coordination. This pilot phase, closely monitored by stakeholders, allowed for the assessment and refinement of the framework, ensuring its continuous improvement and relevance for enhancing healthcare resilience.

To discern the characteristics contributing to enhancing resilience, we conducted a thorough analysis, referring to the resilience-related literature. We identified essential characteristics that must be continuously present within healthcare systems to ensure resilience during crises, such as having a surveillance system capable of monitoring.

However, we acknowledged that some characteristics need to be enhanced only during the occurrence of shocks, varying depending on the specificities of each healthcare system and the consequences of the shocks. This distinction is crucial in the decision-making process, answering questions like, “Does the system need a change in capacities?” This realization is what we aim to bridge from conceptual to heuristic and integrate.

We operationalized the identified characteristics of resilience in healthcare systems by maintaining a balance between constant and contingent features. The continuous characteristics are those perpetually present in the system, while the contingent ones are adjusted based on the conditions and specificities of the system, with the aid of decision-makers.

This methodological approach, focusing on both constant and contingent resilience characteristics, fosters a more nuanced understanding of healthcare system resilience. It integrates the theoretical insights from the literature with practical, heuristic applications, allowing for a more tailored and effective implementation of resilience-enhancing strategies in diverse healthcare settings. Through this integration, we aim to create a framework that is both academically rigorous and practically applicable, ensuring the adaptability and relevance of our model in enhancing healthcare resilience.

**Figure 1:**
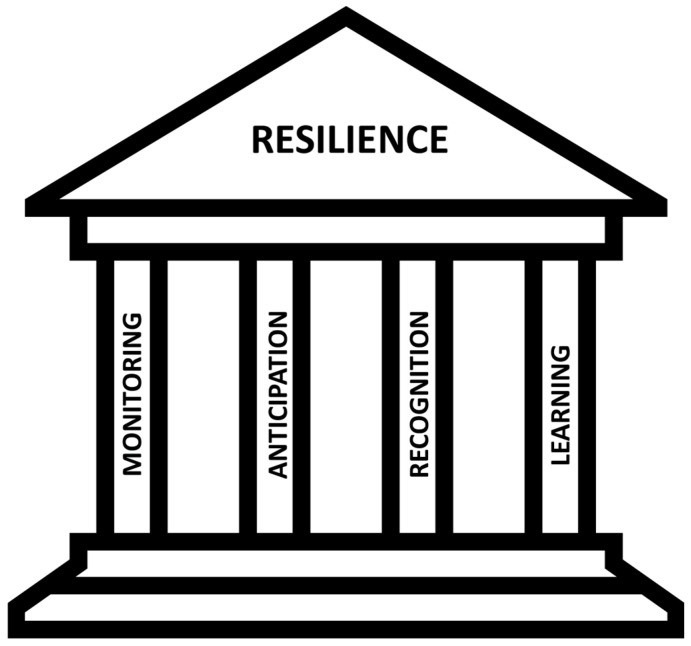
Four cornerstones of resilience at critical infrastructures.

Drawing inspiration from Holling’s seminal resilience framework for critical infrastructures, we have architecturally structured our framework [32]. This schema is intricately anchored upon four cornerstone principles: monitoring, anticipation, recognition, and learning (Figure 1).

Monitoring encompasses the system’s capacity to oversee and govern its current status, environmental factors, information flow, and interdependencies. This capability is crucial for enhancing its situational assessment and awareness. Anticipation represents the system’s foresight regarding environmental impacts, predictions of system behavior, and the simulation and assessment of challenges it might face when confronted with a shock. Recognition denotes the system’s adeptness in identifying ongoing and emerging crises, along with internal or environmental changes. This ability aids in assessing the risk of potential catastrophes, providing essential input for operational decision-making. Finally, learning entails acquiring knowledge from reliable crisis recognition, exploring crisis management possibilities, suitable countermeasures, and decision support. This process equips the system with the skills, means, and measures required for operational resilience management.

**Figure 2:**
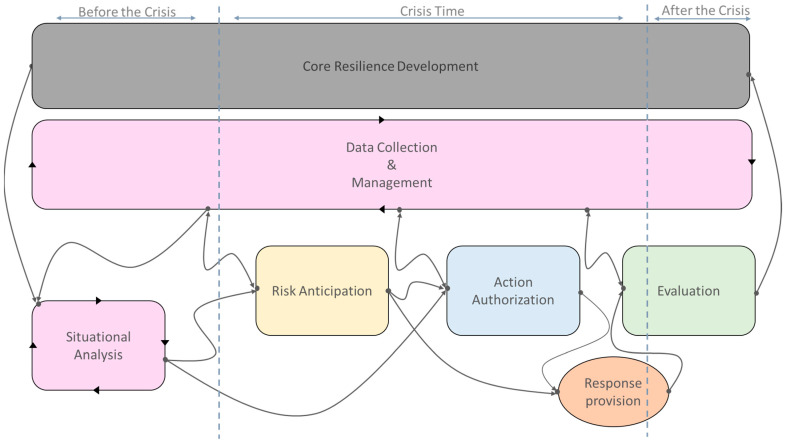
Resilience framework for healthcare systems.

In Figure 2, the healthcare system resilience framework, crafted in accordance with these four core pillars, is depicted.

## 4. Discussion

### 4.1. Overview

Initiating the development of the framework necessitates delineating the resilience process within the healthcare system. This framework is conceptualized based on process-oriented research, underscoring the dynamic interplay between resilience, the healthcare system, and the surrounding environment [33]. Furthermore, crisis management entails efficacious responses to unforeseen events, not solely post-event but also during and pre-event [34]. In light of this, we incorporate these three temporal phases in defining our stages.

Furthermore, it is noteworthy that the boundaries of the healthcare system are explicitly delineated and determined principally by its components, functions, resources, and limitations. The proposed framework aids in identifying events that could disrupt the health system, either predictively or reactively, and facilitates decision-making based on the system’s status to proffer appropriate coping strategies.

### 4.2. Core Resilience Development

**Figure 3:**
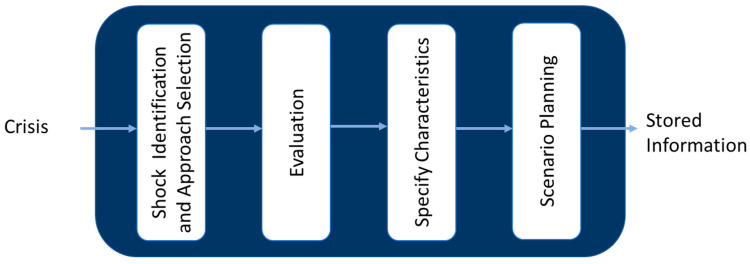
Flow chart of core resilience development.

Core resilience development represents a fundamental and indispensable progression towards bolstering resilience within the healthcare system. This process necessitates the meticulous management and refinement of the inherent capabilities and distinctive characteristics that have previously fortified the system amidst crisis scenarios (Figure 3).

The core resilience development stage scrutinizes the strategies previously employed by the healthcare system to withstand crises, encapsulating them within frameworks such as scenario planning or crisis management plans. The accumulations of the experiences, information, knowledge, capabilities, scenarios, and management plans garnered by the healthcare system during times of crisis and assessed after the crisis serve as adaptable resources for analogous situations in the future, subject to necessary modifications [35,36].

Nevertheless, the healthcare system encounters obstacles when confronted by an unprecedented threat manifesting unique characteristics, frequently necessitating novel adaptations or expansions. Under such circumstances, it becomes imperative to ascertain that the existing data and information continue to fulfill the requisites emanating from the altered condition [37,38]. Consequently, the core resilience development must be delineated by the characteristics of the shocks, the objectives, and the issues pertinent to the intended resilience management.

The findings derived from this segment are subsequently integrated during the stages of ‘Situation Analysis’ and ‘Crisis Management’.

### 4.3. Data Collection and Management

Data collection and situation analysis are steps that must be performed simultaneously and repetitively. These two tasks are related to the monitoring capability in the healthcare system and the environment [6,39].

**Figure 4:**
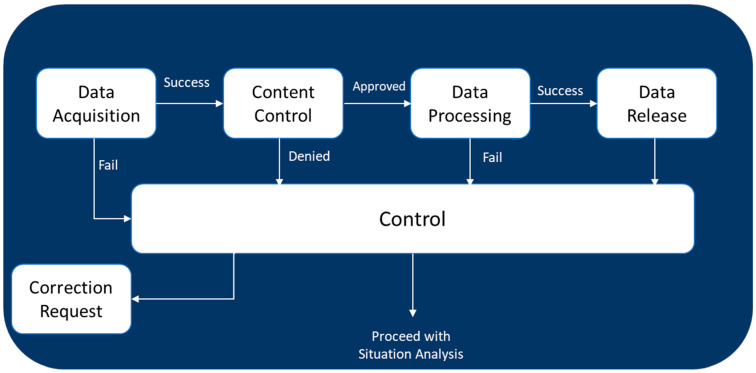
Flow chart of data collection and management.

The primary aim of data collection and management is to identify and gather the requisite data and information essential for the concurrent task of situation analysis [39]. It is imperative to verify the quality, content, and availability of validation after the acquisition of data [40,41]. Any failures or deviations encountered during data collection and processing must be promptly reported to the control system [12,14]. This diligent approach ensures the integrity and reliability of the information, thereby underpinning a robust framework for situation analysis (Figure 4).

Given the dynamic nature of the healthcare system, there is an inherent necessity to amass a diverse array of data, encompassing performance indicators, the service quality, environmental factors, hazards, experiential learnings, capacities, resources, and the like. Occasionally, there emerges a requisite to amalgamate this data to garner insights of a higher echelon, such as discerning cause-and-effect relationships, root causes, etc. The presence of faults or flaws within the data collection system, or the suboptimal quality of the acquired data, precipitates the failure of data processing, necessitating detection and rectification by the system. Ultimately, upon the successful execution of data collection and processing, the resultant insights are furnished for subsequent stages and further actions [20,42].

### 4.4. Situation Analysis

**Figure 5:**
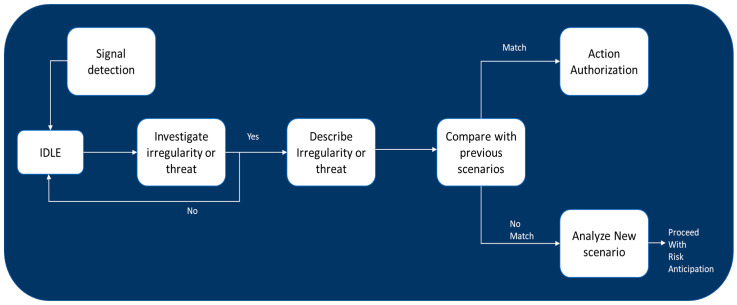
Flow chart of situation analysis.

Situational analysis stands as a cornerstone task within resilience management in healthcare systems (Figure 5). The landscape of situational alterations is subject to continual monitoring and analysis, with the objective of discerning and scrutinizing conditions being deemed critical. The inception of situational analysis is marked by the identification of early indicators signaling the emergence of potential threats.

Signal detection embodies the methodology of identifying early warning signs or indicators that preclude a potential crisis prior to its full manifestation. It is imperative for a healthcare system to adeptly discern these signals, as this capability facilitates the initiation of proactive measures and the activation of a response plan designed to attenuate the impact of a crisis [2]. For effective signal detection within a resilient healthcare system, there is a requisite for a comprehensive surveillance system capable of monitoring multifarious real-time data and assessing them for potential warning indications. The selection of detection methods can be influenced by the nature and complexity of the available data, along with the objectives of resilience management in diverse healthcare systems [8,23,43].

The situational analysis returns to normal status if no anomaly is observed during this phase. But if the system detects any abnormality or threat, it needs to describe it in detail and its characteristics. The system must next compare the detected irregularities to any probable state in the earlier scenarios to determine which possibilities best describe the current situation. If the state of the current situation matches the previous scenarios in the system, starting risk anticipation is unnecessary. This helps to prevent an unnecessary analysis to determine potential risks and issues. New scenarios must be analyzed for sufficient details to provide a well-formulated problem description for further investigation in the following stages [12,14,44].

### 4.5. Risk Anticipation

The crisis management group does the risk anticipation and action authorization (next step) according to the specific situation of each healthcare system, the objectives, limitations, and constraints. This is an advantage of this framework that makes the framework adaptable enough to be implemented in diverse healthcare systems with various characteristics.

In a crisis, the health system may undergo negative changes and face different pressures that increase the risk in several aspects such as a treatment system interruption or a decreasing in the quality of services; treatment errors due to a lack of information, cognitive errors, exhaustion, or fatigue; life-threatening incidents due to exposure to hazardous situations; lack of resources (human, medicine, infrastructure, etc.).

**Figure 6:**
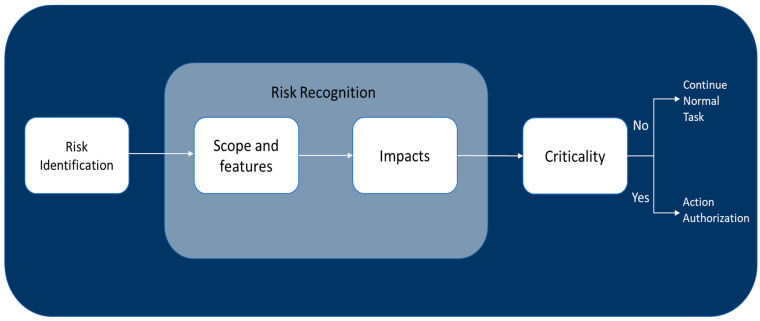
Flow chart of risk anticipation.

The healthcare system must be analyzed regarding the potential risks linked to threat expansion in the face of a new scenario or threat [45]. Then, the scope and features of the risk such as its severity, corresponding likelihood and feasible short-term development are investigated (Figure 6).

In the next step, all achieved results are analyzed regarding the healthcare system performance indicators to understand the impact of the corresponding risk on the system. Finally, it is determined whether the risk is critical based on the information obtained. If the system does not detect any criticality, it keeps working on the regular tasks; nevertheless, if it does, it continues to approve some resilience management measures [46,47,48].

### 4.6. Action Authorization

Each healthcare system is obliged to make cautious decisions grounded on the unique features, imperatives, and demands it discerns amidst a crisis. These decisions, fundamentally, are organizational actions that health systems need to take to enhance their resilience and effectively manage shocks and crises. Resilience characteristics embody organizational measures that, with proper implementation or enhancement, significantly boost resilience. This approach ensures that healthcare systems are primed to navigate the complexities of challenges and disruptions efficiently.

**Figure 7:**
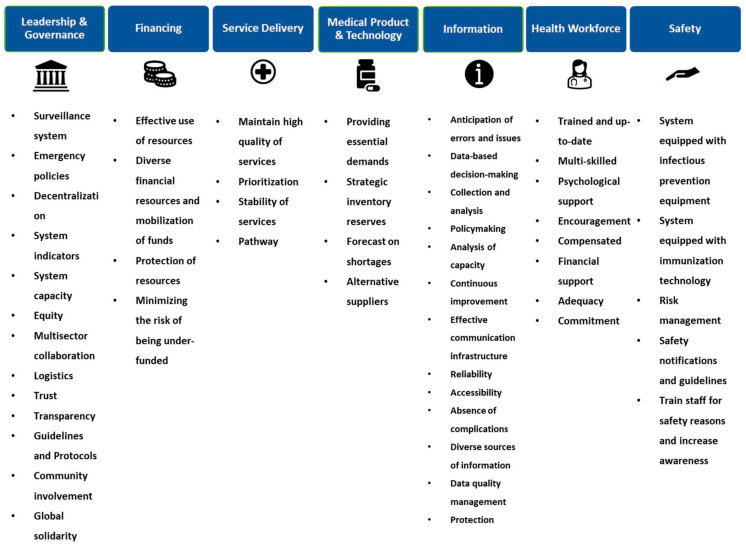
Healthcare system resilience characteristics.

Recent research in this domain has led to a wealth of recommendations for such actions, with empirical evidence underscoring the effectiveness of certain actions in enabling systems to absorb, adapt, or transform in response to shocks [31]. This body of work, summarized visually in Figure 7, offers a comprehensive guide for healthcare systems seeking to adopt strategies proven to enhance crisis management capabilities.

However, the simultaneous strengthening and focusing on all identified characteristics during shocks or crises proves to be an impractical endeavor due to the variability in time, resources, and demands. This realization underscores the necessity for health systems to engage in ‘action authorization’, making optimal decisions based on their specific contexts. By tailoring their strategies to meet particular needs and leveraging available opportunities, health systems can navigate crises more adeptly, prioritizing actions that align with their unique features and constraints.

These determinations inherently vary across diverse systems, thereby precluding the possibility of establishing a universal guideline or protocol applicable to all systems or departments. In this context, it is pivotal to consider the multifarious characteristics delineated in the existing literature (Figure 7) aimed at augmenting the efficacy and resilience of healthcare systems [31].

Drawing insights from previous phases and recognizing the unique needs and attributes of each sector, decision-makers in healthcare systems bear a crucial responsibility. Each system, with its distinct objectives, constraints, identified risks, geographic considerations, and governing rules, requires tailored strategies to effectively manage crises. Empowered with the necessary authority, decision-makers must prioritize and reinforce distinctive features, ensuring adaptability and responsiveness to evolving challenges [47,49].

Adopting a nuanced and personalized approach to resilience management, supported by decision science and cost–benefit analysis, allows healthcare systems to address their specific demands efficiently, conserving time, energy, and resources. This approach negates the need for more rigid, one-size-fits-all frameworks and protocols, thereby enhancing adaptability.

By embracing a holistic strategy that considers the diversity and intricacies inherent in different healthcare segments, a more robust and adaptable framework can be cultivated. This framework is vital for navigating the multifaceted challenges encountered, ultimately enhancing the overall effectiveness and sustainability of healthcare systems across varied contexts. The harmonious integration of a nuanced understanding of system-specific needs with authorized, informed action is essential for optimizing each system’s ability to manage emergent healthcare challenges.

### 4.7. Evaluation

**Figure 8:**
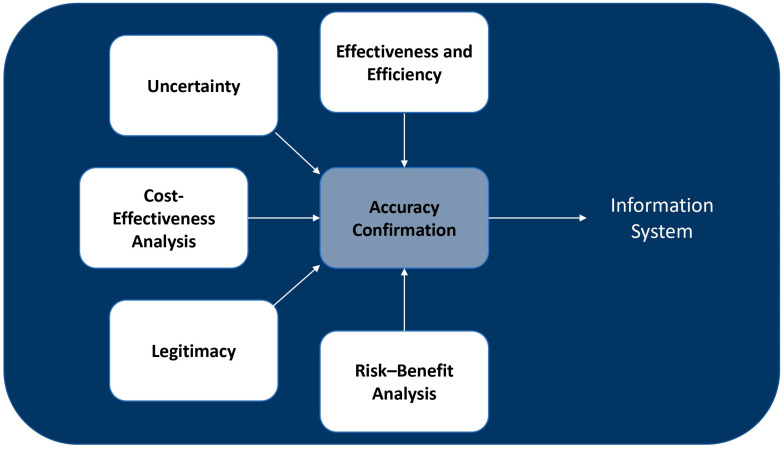
Flow chart for the evaluation stage.

The final stage entails a thorough evaluation of the authorized interventions implemented to address the identified crises, along with an assessment of their outcomes, as illustrated in Figure 8. The goal is to formulate strategies to mitigate similar threats in the future. This assessment process commences during a crisis, immediately following the authorization and implementation of actions, and extends beyond the crisis period to examine both the short-term and long-term effects. Given the varied impacts of decisions and actions, a multidimensional evaluation approach is essential for healthcare systems. This approach ensures a comprehensive understanding of the effects of interventions and informs the development of improved strategies for future challenges.

The effectiveness and efficiency of the decisions made about the expanding threat are first examined using the data and information obtained from the monitoring system. Moreover, the uncertainty arising from different sources about authorized actions is evaluated to determine the validity of decisions. The healthcare system must additionally conduct a cost-effectiveness analysis. This is an important tool for the healthcare system as it offers objective data regarding costs, resource utilization, shortages, and outcomes [45,49,50]. Furthermore, given that the health system is founded on human interactions, decisions must be legitimate to be accepted by those working in the system and the patients who receive services. Finally, a risk–benefit analysis helps evaluate the risk and benefits of decisions, actions, and procedures to deal with the crisis or provide alternative suggestions. After confirming accuracy, the evaluation phase results are recorded in the information system.

## 5. Conclusions

In this research, we have developed a new framework to significantly enhance the resilience of healthcare systems. By advancing beyond existing crisis management models, this framework is specially tailored for a comprehensive analysis of healthcare systems. We have identified a cyclical pattern of resilience enhancement within these systems, analyzing not only the time of the crisis but also the precursors and aftermath of each crisis. Our framework aims to provide actionable strategies at every stage of this cycle, enhancing our understanding of each incident’s resolution, anticipating future events, and enriching our knowledge with insights from previous occurrences.

Decision-making within the framework introduced in our study is based on key resilience-enhancing characteristics. At the heart of this model, the characteristics are organized into seven foundational building blocks, designed to facilitate its usage, and inform decision-making processes. This approach allows for the adoption of various decisions that are uniquely tailored to the specific conditions and requirements of the system. Essentially, these characteristics guide the implementation strategies that enable the system to adapt and maintain its performance in the face of crises or shocks. By strategically integrating these elements into the different sectors of the healthcare system—each with distinct features—we aim to foster resilience in a manner that is finely tuned to their particular needs. This methodology effectively bridges the gap between theoretical concepts and practical application, significantly minimizing the risk of implementation failures. 

## Data Availability

No new data were created or analyzed in this study. Data sharing is not applicable to this article.
